# Preoperative selpercatinib induces major pathological response in a patient with stage IIIA, RET fusion-positive non-small cell lung cancer: A case report

**DOI:** 10.1097/MD.0000000000045554

**Published:** 2025-10-31

**Authors:** Kristina Breitenecker, Hannah Fabikan, Christoph Weinlinger, Dagmar Krenbek, Thomas Klikovits, Stefan Watzka, Arschang Valipour, Maximilian Johannes Hochmair

**Affiliations:** a Karl Landsteiner Institute for Lung Research and Pulmonary Oncology, Klinik Floridsdorf, Vienna, Austria; b Department of Pathology, Klinik Floridsdorf, Vienna Healthcare Group, Vienna, Austria; c Department of Thoracic Surgery, Klinik Floridsdorf, Vienna Healthcare Group, Vienna, Austria; d Karl Landsteiner Institute for Clinical and Translational Thoracic Surgery Research, Klinik Floridsdorf, Vienna, Austria; e Paracelsus Medical University, Salzburg, Austria; f Department of Respiratory and Critical Care Medicine, Klinik Floridsdorf, Vienna Healthcare Group, Vienna, Austria.

**Keywords:** case report, major pathological response, non-small cell lung cancer, preoperative therapy, selpercatinib

## Abstract

**Rationale:**

The success of tyrosine kinase inhibtors in the neoadjuvant and adjuvant setting provides a rationale for exploring selpercatinib in the perioperative setting to improve patient outcomes in early-stage lung cancer. However, selpercatinib is currently only approved as first-line treatment for advanced rearrangement during transfection (RET) fusion-positive non-small cell lung cancer (NSCLC), limiting its use and potential benefit for earlier stage patients. Hence, this case report explores selpercatinib’s potential use in early-stage disease to address this treatment gap and improve patient outcomes.

**Patient concerns:**

We report the case of a 75-year-old female with no history of smoking who was referred to our institute with a chest computed tomography scan revealing a mass in the right lower lung lobe.

**Diagnoses:**

The patient was diagnosed with RET fusion-positive lung adenocarcinoma based on findings from radiologic findings from computed tomography (CT) and positron emission tomography-CT scans, histopathological examination, and molecular profiling through next-generation sequencing.

**Interventions:**

The patient was treated with 4 cycles of carboplatin and pemetrexed. A follow-up CT scan revealed the progression of the mediastinal lymph node, leading to the decision to initiate selpercatinib treatment. Due to a major size reduction of the primary lesion and the absence of tumor cells in the lymph nodes, the patient underwent curative lobectomy and lymphadenectomy.

**Outcomes:**

Preoperative selpercatinib induced tumor regression, enabling surgical resection. Histopathological examination of the resected tumor revealed a major pathological response to preoperative selpercatinib treatment.

**Lessons:**

This case highlights the potential of selpercatinib in the perioperative setting, suggesting that it induces tumor regression and enabling of curative surgery in early-stage RET fusion-positive NSCLC. Our findings support the further investigation of selpercatinib in early stages and provide a rationale for future treatment strategies of early-stage RET fusion-positive NSCLC patients.

## 1. Introduction

Fusions of the rearrangement during transfection (RET) gene present in 1% to 2% of all non-small cell lung cancer (NSCLC) patients,^[1]^ resulting in constitutive activation of oncogenic signaling pathways, promoting tumor growth and survival. RET fusions are more commonly found in younger patients with adenocarcinoma histology and minimal to no smoking history.^[2]^ Selpercatinib, an ATP-competitive, highly selective, small molecule inhibitor of the RET kinase, has significantly improved outcomes of patients harboring this genomic alteration.^[1]^ Unlike previously used multiple-tyrosine kinase inhibitors, selpercatinib displays a high selectivity for RET, thus reduced off-target toxicity as well as increased intracranial activity.^[3]^ The LIBRETTO-001 trial demonstrated the safety and efficacy of selpercatinib in advanced NSCLC patients, which granted its US Food and Drug Administration approval for that indication in 2020.^[1]^ In addition, selpercatinib has demonstrated superior efficacy over platinum-based chemotherapy with or without pembrolizumab in the first-line treatment of RET fusion-positive advanced stage NSCLC patients.^[4]^ While other targeted therapies, such as epidermal growth factor receptor (EGFR) and anaplastic lymphoma kinase inhibitors, are actively investigated in the neoadjuvant setting, and have shown promising results in the adjuvant setting, selpercatinib is currently limited for the treatment of advanced stages and has not been systematically tested in the neoadjuvant setting.^[5–8]^

Hence, we present a case of an RET fusion-positive, stage IIIA NSCLC patient who progressed on chemotherapy and subsequently received preoperative selpercatinib treatment, achieving major pathological response (MPR). This case highlights the potential of selpercatinib in the perioperative setting and provides a rationale for future treatment strategies of early-stage RET fusion-positive NSCLC patients.

## 2. Case report

A 75-year-old female with no history of smoking was referred to our institute with a chest computed tomography (CT) scan revealing a mass in the right lower lung lobe. Evaluation of the CT scan showed a 25 × 35 mm lesion, including mediastinal and hilar lymphadenopathy. A CT-guided core needle biopsy of the suspected mass was done and analyzed, revealing the presence of a TTF-1 positive, moderately differentiated pulmonary adenocarcinoma. [^18^F] Fluorodeoxyglucose–positron emission tomography confirmed the primary tumor mass including the involvement of mediastinal and hilar lymph nodes but excluded metastatic seeding elsewhere. Based on the radiologic evaluation, the patient was diagnosed with stage IIIA (T2a N2 M0) pulmonary adenocarcinoma. Molecular profiling using next-generation sequencing (AmpliSeq for Illumina Focus Panel and AmpliSeqTM Library PLUS for Illumina; MiniSeqTM system; Illumina, San Diego) determined a *KIF5B-RET* fusion in the absence of any other accompanying genomic aberrations.

Given the tumor staging, treatment with 4 cycles of carboplatin (AUC5) and pemetrexed (500 mg/m²) was initiated, which resulted in regression of the primary tumor mass from 27 × 35 mm to 17 × 27 mm at the follow-up CT at 3 months following diagnosis. Despite a partial response of the primary lesion and size stable hilar lymph nodes, there was a progression of the mediastinal lymph node in position 4R from 32 × 36 mm to 35 × 40 mm. Therefore, the patient did not proceed with curative surgery. The patient case was discussed at the multidisciplinary board meeting, resulting in unanimous recommendation to initiate (off-label) selpercatinib treatment, with a regimen of 1 × 160 mg twice daily. Overall, the treatment was well tolerated, with the exception of the patient experiencing grade 3 hyponatremia, which led to a dose reduction to 80 mg twice daily.

**Figure 1.**
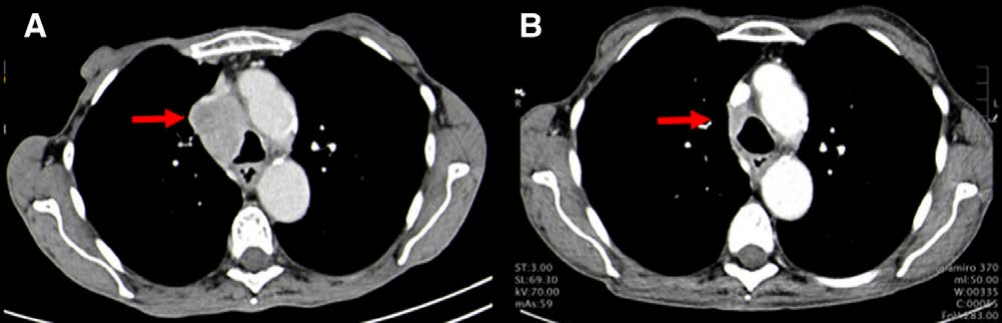
Radiologic response to selpercatinib. (A) CT scan of the chest post 4 cycles of chemotherapy and prior to selpercatinib initiation, revealing a large mediastinal lymph node in position 4R (red arrow). (B) Follow-up CT scan of the chest showing substantial lymph node regression (red arrow) after 2 months of neoadjuvant selpercatinib treatment. CT = computed tomography.

**Figure 2.**
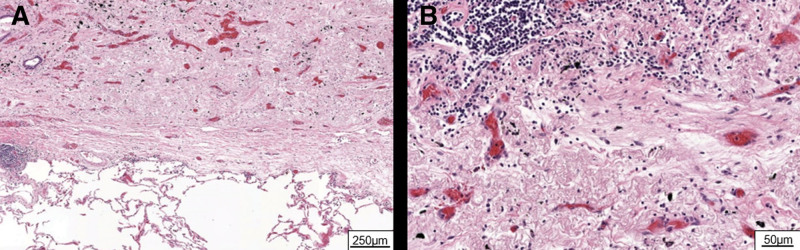
MPR induced by preoperative selpercatinib. (A, B) Hematoxylin and eosin (H&E) stainings of resected primary tumor lesion post selpercatinib treatment. (A) Image showing the tumor bed with fibroelastotic scar tissue and adjacent normal lung parenchyma (5× magnification). (B) Tumor bed with fibroelastotic scar tissue, fibroelastosis, and lymphocytes (20× magnification). MPR = major pathological response.

Within 2 months of selpercatinib initiation, a follow-up CT scan determined a major size reduction of the mediastinal lymph node and no changes in size of the primary lesion (Fig. 1). To determine the subsequent treatment approach, the mediastinal lymph nodes were biopsied for reevaluation using endobronchial ultrasound-guided transbronchial needle aspiration. Cytological and histologic analysis of the lymph node confirmed the absence of malignancy. Therefore, the patient underwent curative lobectomy and lymphadenectomy. Histologic evaluation of the resected primary tumor revealed an MPR grade IIb according to the classification by Junker^[9]^ (Fig. 2). The tumor mass made up of 75% fibrotic tissue, 17% of inflammatory cells, 8% vital tumor cells, and 0% necrosis. Accordingly, the patient was restaged as ypT1b ypN0 R0. The patient is planned to continue adjuvant selpercatinib treatment up to 24 months.

## 3. Discussion

We report a case of a stage IIIA, RET fusion-positive NSCLC patient who achieved MPR after treatment with selpercatinib in a preoperative setting.

While targeted therapies are standard-of-care in advanced, oncogene-addicted NSCLC patients, the therapeutic landscape of early-stage patients primarily consists of local therapies in combination with chemo and/or immunotherapy.^[10,11]^ The LIBRETTO-432 trial is currently investigating the safety and efficacy of adjuvant selpercatinib versus placebo in stage IB-IIIA, RET fusion-positive NSCLC patients to address this issue.^[12]^ In support of this trial, recent phase-III clinical trials, such as the ALINA and ADAURA trials, have already demonstrated the efficacy of adjuvant anaplastic lymphoma kinase and EGFR-targeted therapies in early-stage lung cancer patients.^[6,13]^

Given the success of improving disease and recurrence-free survival in these studies, novel trials are examining the use of targeted therapies in the neoadjuvant setting. The NEOADAURA trial investigates the efficacy and safety of neoadjuvant osimertinib as monotherapy or in combination with chemotherapy in resectable EGFR-mutant lung cancer patients.^[8]^ With the exception of the cohort 7 of the LIBRETTO-001 phase 1-2 trial, there are currently no trials investigating the efficacy of neoadjuvant Selpercatinib in lung cancer patients.^[14]^ Hence, our case underlines the potential of preoperative selpercatinib in improving resectability in early-stage RET fusion-positive NSCLC and provides a rationale for employing selpercatinib in early stages, too. In support of our findings, another case report described a patient, participating in the LIBRETTO-001 trial, who achieved a pathological complete response after neoadjuvant selpercatinib treatment.^[15]^

In addition to NSCLC patients, cases of RET fusion-positive medullary thyroid cancer and sarcoma patients experiencing tumor regression with neoadjuvant selpercatinib treatment have been reported, which further underline the rationale to implement selpercatinib in early stages.^[16–18]^

The LIBRETTO-431 trial showed that selpercatinib had a higher incidence of adverse events compared with standard-of-care chemotherapy with or without pembrolizumab.^[4]^ Selpercatinib-related AEs included increased liver transaminases, hypertension, diarrhea, and edema. In this case, selpercatinib was well tolerated but the patient experienced an event of hyponatremia leading to a dose reduction. In line with this case, dose reductions due to adverse events occurred in 51% of selpercatinib-treated patients compared with the control group. While the incidence of adverse events (any grade and grade ≥ 3) with selpercatinib was higher compared with standard-of-care chemo/immunotherapy, it demonstrated superior efficacy, providing a strong rationale for employing selpercatinib in RET fusion-positive patients.^[4]^

A limitation to this case report is the absence of a histological analysis, determining the tumor load after completion of chemotherapy and prior to selpercatinib initiation. This analysis could provide a more detailed insight into this treatment regimen and would potentially strengthen our rationale. However, based on the radiologic findings, indicating disease progression, taking an additional biopsy was refrained from. An additional, potential limitation is the lack of a positron emission tomography-CT after selpercatinib initiation and prior to surgical resection, resulting in the use of different imaging modalities. However, a biopsy was performed, which confirmed the absence of malignancy in the lymph nodes. Additionally, the follow-up period from selpercatinib initiation is relatively short. Nonetheless, this does not diminish the significance of our findings as preoperative selpercatinib treatment induced sufficient tumor regression to enable curative surgery.

## 4. Conclusion

In conclusion, we present a case of an RET fusion-positive NSCLC patient who achieved an MPR within 2 months of preoperative selpercatinib treatment. In detail, the patient exhibited a major response to preoperative selpercatinib treatment. Imaging demonstrated a major size reduction of the primary tumor lesion and complete response of the lymph nodes enabling the patient for surgical resection. Subsequent histopathological examination of the resected tumor revealed an MPR with minimal residual viable tumor cells present. To the best of our knowledge, this is the first case of a patient treated in the neoadjuvant setting outside of a clinical trial. Hence, this case contributes to the growing body of evidence, which supports the use of targeted therapies, specifically selpercatinib, to improve the outcomes of early-stage NSCLC patients, both in the neoadjuvant and adjuvant settings.

## Author contributions

**Conceptualization:** Maximilian Johannes Hochmair.

**Formal analysis:** Dagmar Krenbek.

**Investigation:** Dagmar Krenbek, Thomas Klikovits, Stefan Watzka, Arschang Valipour, Maximilian Johannes Hochmair.

**Methodology:** Dagmar Krenbek.

**Project administration:** Hannah Fabikan, Christoph Weinlinger.

**Resources:** Thomas Klikovits, Stefan Watzka, Arschang Valipour, Maximilian Johannes Hochmair.

**Supervision:** Hannah Fabikan, Christoph Weinlinger, Maximilian Johannes Hochmair.

**Writing – original draft:** Kristina Breitenecker.

**Writing – review & editing:** Hannah Fabikan, Christoph Weinlinger, Thomas Klikovits, Arschang Valipour, Maximilian Johannes Hochmair.

## References

[1] Drilon A Oxnard GR Tan DSW. Efficacy of selpercatinib in RET fusion-positive non-small-cell lung cancer. N Engl J Med. 2020;383:813–24. 32846060 10.1056/NEJMoa2005653 PMC7506467

[2] Drilon A Hu ZI Lai GGY Tan DSW. Targeting RET-driven cancers: lessons from evolving preclinical and clinical landscapes. Nat Rev Clin Oncol. 2018;15:151–67. 29134959 10.1038/nrclinonc.2017.175 PMC7938338

[3] Drilon A Rekhtman N Arcila M. Cabozantinib in patients with advanced RET-rearranged non-small-cell lung cancer: an open-label, single-centre, phase 2, single-arm trial. Lancet Oncol. 2016;17:1653–60. 27825636 10.1016/S1470-2045(16)30562-9 PMC5143197

[4] Zhou C Solomon B Loong HH. First-line selpercatinib or chemotherapy and pembrolizumab in RET Fusion-Positive NSCLC. N Engl J Med. 2023;389:1839–50. 37870973 10.1056/NEJMoa2309457 PMC10698285

[5] Tsuboi M Herbst RS John T. Overall survival with osimertinib in resected EGFR-mutated NSCLC. N Engl J Med. 2023;389:137–47. 37272535 10.1056/NEJMoa2304594

[6] Wu YL Dziadziuszko R Ahn JS. Alectinib in resected ALK-positive non-small-cell lung cancer. N Engl J Med. 2024;390:1265–76. 38598794 10.1056/NEJMoa2310532

[7] Leonetti A Minari R Boni L. Phase II, open-label, single-arm, multicenter study to assess the activity and safety of alectinib as neoadjuvant treatment in surgically resectable stage III ALK-positive NSCLC: ALNEO Trial. Clin Lung Cancer. 2021;22:473–7. 33762169 10.1016/j.cllc.2021.02.014

[8] Tsuboi M Weder W Escriu C. Neoadjuvant osimertinib with/without chemotherapy versus chemotherapy alone for EGFR-mutated resectable non-small-cell lung cancer: NeoADAURA. Future Oncol. 2021;17:4045–55. 34278827 10.2217/fon-2021-0549 PMC8530153

[9] Junker K Thomas M Schulmann K Klinke V Bosse U Muller KM. [Regression grading of neoadjuvant non-small-cell lung carcinoma treatment]. Pathologe. 1997;18:131–40. 9244871 10.1007/s002920050201

[10] Hendriks LE Kerr KM Menis J. Oncogene-addicted metastatic non-small-cell lung cancer: ESMO clinical practice guideline for diagnosis, treatment and follow-up. Ann Oncol. 2023;34:339–57. 36872130 10.1016/j.annonc.2022.12.009

[11] Remon J Soria JC Peters S. Early and locally advanced non-small-cell lung cancer: an update of the ESMO clinical practice guidelines focusing on diagnosis, staging, systemic and local therapy. Ann Oncol. 2021;32:1637–42. 34481037 10.1016/j.annonc.2021.08.1994

[12] Tsuboi M Goldman JW Wu YL. LIBRETTO-432, a phase III study of adjuvant selpercatinib or placebo in stage IB-IIIA RET fusion-positive non-small-cell lung cancer. Future Oncol. 2022;18:3133–41. 35950566 10.2217/fon-2022-0656

[13] Wu YL Tsuboi M He J. Osimertinib in resected EGFR-mutated non-small-cell lung cancer. N Engl J Med. 2020;383:1711–23. 32955177 10.1056/NEJMoa2027071

[14] Rajaram R Sholl LM Dacic S. LIBRETTO-001 cohort 7: a single-arm, phase 2 study of neoadjuvant selpercatinib in patients with resectable stage IB-IIIA <i>RET</i> fusion-positive NSCLC. J Clin Oncol. 2022;40(16_suppl):TPS8594–TPS8594.

[15] Goldman JW Sholl LM Dacic S. Case report: complete pathologic response to neoadjuvant selpercatinib in a patient with resectable early-stage RET fusion-positive non-small cell lung cancer. Front Oncol. 2023;13:1178313. 37274265 10.3389/fonc.2023.1178313 PMC10232990

[16] Jozaghi Y Zafereo M Williams MD. Neoadjuvant selpercatinib for advanced medullary thyroid cancer. Head Neck. 2021;43:E7–E12. 33169506 10.1002/hed.26527 PMC7756223

[17] Pitoia F Abelleira E Roman-Gonzalez A. Neoadjuvant treatment of locally advanced thyroid cancer: a preliminary latin American experience. Thyroid. 2024;34:949–52. 38757613 10.1089/thy.2024.0090

[18] Schrenk KG Weschenfelder W Spiegel C. Exceptional response to neoadjuvant targeted therapy with the selective RET inhibitor selpercatinib in RET-fusion-associated sarcoma. J Cancer Res Clin Oncol. 2023;149:5493–6. 36469155 10.1007/s00432-022-04496-y PMC10356868

